# Longitudinal reference centiles for the Gross Motor Function Measure‐66 in children and adolescents with cerebral palsy

**DOI:** 10.1111/dmcn.16455

**Published:** 2025-08-05

**Authors:** Ibrahim Duran, Leonie Schafmeyer, Bruno Lentzen, Karoline Spiess, Titus Keller, Kyriakos Martakis, Eckhard Schoenau

**Affiliations:** ^1^ University of Cologne, Medical Faculty and University Hospital Center of Prevention and Rehabilitation UniReha Cologne Germany; ^2^ Department of Pediatrics University of Cologne, Medical Faculty and University Hospital Cologne Germany; ^3^ Department of Pediatric Neurology Justus‐Liebig‐University Giessen Giessen Germany

## Abstract

**Aim:**

To establish novel longitudinal reference values for the Gross Motor Function Measure‐66 (GMFM‐66) in children and adolescents with cerebral palsy aged 3 to 18 years, to enable more accurate assessments of changes in motor function.

**Method:**

This was a single‐centre retrospective analysis of patients who participated in a rehabilitation programme between January 2006 and March 2022. The GMFM‐66 was used to measure gross motor function. Paired GMFM‐66 measurements from the follow‐up phase of the rehabilitation programme were used to establish a reference centile for the change in GMFM‐66 over a 6‐month period using the lambda–mu–sigma method.

**Results:**

Reference centiles for GMFM‐66 changes (over a 6‐month period; ±1 month) were created using 1190 longitudinal data pairs of GMFM‐66 measurements (mean age 8 years 4 months [standard deviation 7 years 11 months] at start of follow‐up), Gross Motor Function Classification System levels I to V. The z‐scores for GMFM‐66 change of a validation dataset by the new tool and the previously described method to quantify a change in GMFM‐66 by individual effect size were highly correlated (Pearson's rank correlation coefficient 0.981 [95% confidence interval 0.979–0.984], *p* < 0.001)

**Interpretation:**

The new reference values showed a high correlation with the previously published reference values, which were limited to an age range of 3 to 12 years. The new reference values can be applied from an age of 3 to 18 years. This facilitates the evaluation of medical treatment after a 6‐month period also for children with cerebral palsy who are older than 12 years.

## Graphical abstract

(a) Shows a three‐dimensional diagram of the mean GMFM‐66 score difference (after 6 month) as a function of the patient's age (in years) and the initial value of the GMFM‐66 score. (b) Shows a three‐dimensional diagram of the standard deviation of the GMFM‐66 score difference as a function of the patient's age (in years) and the initial value of the GMFM‐66 score.

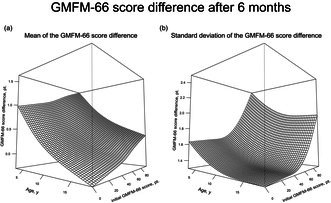

**This original article is commented on by Rosenbaum on pages** 153–154 **of this issue.**

**What this paper adds**

- Novel longitudinal Gross Motor Function Measure‐66 (GMFM‐66) reference centiles for children with cerebral palsy aged 3 to 18 years are established.
- The z‐scores for a GMFM‐66 score difference over a 6‐month period can be calculated.
- The z‐scores are specific for age and initial GMFM‐66 score.

**Abbreviations**
GMFM: Gross Motor Function Measure
iES: individual effect size

Cerebral palsy (CP) is the most common movement disorder in childhood, the aetiology of which is a non‐progressive lesion in the developing brain.^1^, ^2^ Clinical findings consist of various symptoms such as spasticity, dyskinesia, and ataxia that affect posture or movement.^2^ The prevalence of CP is 2 to 3 per 1000 live births according to the Surveillance of Cerebral Palsy in Europe.^3^

Children with CP require functional therapy. The aim of therapy is to minimize sequelae, such as skeletomuscular complications and to promote development that improves participation in daily life.^4^ It is essential to evaluate the impact of therapy regularly to justify its continuation. For this purpose, a measurement procedure should be performed before the start of therapy as a baseline and after completion of therapy.^5^ A common measurement is the Gross Motor Function Measure (GMFM), which is used to assess functional capacity in a standardized manner.^6^ There are different versions of the GMFM, which differ in the number of test items. The GMFM originally consisted of 88 test items,^6^ but the GMFM‐66 with only 66 items is now the most commonly used gross motor test.^6^, ^7^, ^8^, ^9^

Interpretation of the GMFM‐66 is challenging because children with CP continue to improve their motor skills up to about 12 years of age, even with standard treatment (e.g. physiotherapy twice a week).^5^, ^8^ If a child participates in a rehabilitation programme in addition to standard treatment, the question arises as to whether the improvement in motor skills is due to the additional medical intervention or would have occurred without it.^5^, ^10^ It is helpful to use reference values or centile curves to assess this more accurately and then interpret the results in the clinical setting.^5^, ^8^, ^11^, ^12^, ^13^ Recently, Sanderlin et al.^14^ analyzed the distribution and longitudinal course of motor changes in children with CP by comparing three different, commonly used centile curves from Rosenbaum et al.,^13^ Hanna et al.,^12^ and Duran et al.^5^ All three studies showed agreements in longitudinal progression and can be applied in clinical and scientific settings.^14^ One advantage of the centile curves from Duran et al. is that the individual effect size (iES) can also be determined and therefore the therapy of the individual patient can be better evaluated.^5^ However, the normative data from Duran et al. only addressed ages 2 to 11 years.^5^ In addition, their reference values are based on cross‐sectional and not longitudinal data.^5^ Collecting longitudinal data is essential to better assess the dynamics of motor changes.^14^

Therefore, this study aimed to establish novel reference values from longitudinal data of a study population with CP who participated in a rehabilitation program from January 2006 to March 2022 to assess changes in gross motor function. Specifically, we aimed to use the reference curves for children and adolescents aged 3 to 18 years to calculate z‐scores of the GMFM‐66 change over a 6‐month period.

## METHOD

### Study design

This was a single‐centre retrospective data analysis of children and adolescents with CP. The study population participated in a rehabilitation programme at the Centre for Prevention and Rehabilitation at the University of Cologne, Germany, from January 2006 to March 2022. The rehabilitation programme is described in more detail in Figure S1.^5^ During the follow‐up period, patients received only 30 minutes of physiotherapy approximately twice weekly (standard therapy). Patients repeated the rehabilitation programme up to three times. Mainly because of waiting times, it is only possible to participate in the rehabilitation programme every 2 to 3 years. Participation in the programme was funded by the German healthcare system.

The inclusion criteria were diagnosis of CP, age under 18 years, and written informed consent from the parents/legal guardians. The patients were classified into the following subgroups: bilateral spastic, unilateral spastic, ataxic, dyskinetic, and mixed type CP.^15^ The functionality of the patients was classified according to the Gross Motor Function Classification System (GMFCS) level.^16^ Approval was obtained from the Ethics Committee of the University of Cologne (16–269). A detailed description of the registry is available at www.germanctr.de (DRKS0001131).

### Motor function classification by GMFCS

The GMFCS was used to classify motor function.^7^, ^16^ The GMFCS consists of an ordinal scale with five levels of motor function (I‐V).^7^, ^16^ Children who could walk freely were divided into GMFCS levels I and II. Children in GMFCS levels III and IV could walk with the help of a walking aid and children classified in GMFCS levels IV and V required a wheelchair, possibly with electrical assistance if available.^7^, ^16^ Classification of GMFCS level^7^, ^16^ was recorded by the attending paediatrician or child neurologist.

### Assessment of motor development using the GMFM‐66

The GMFM‐66 is a standardized measuring instrument that is suitable for the objective measurement of motor skills in the dimensions of rolling, crawling and kneeling, sitting, standing and walking. Russell et al. showed a very good reliability of the GMFM‐66.^17^ The GMFM‐66 was assessed at three different times (per year of therapy): in the first year of rehabilitation, at the beginning as baseline (M0); after the 6‐month rehabilitation programme (M6); and at the 6‐month follow‐up (M12). In the second, third, and fourth years, the GMFM‐66 post‐training assessment was performed after 4 months (M4) and the follow‐up was performed 8 months later (M12).^18^ Gross Motor Ability Estimator (version 2) scoring software was used to evaluate the GMFM‐66 score (*CanChild*, McMaster University, Ontario, Canada). Physiotherapy up to approximately twice a week was continued during the programme and the follow‐up intervals as part of the national standard care of children and adolescents with CP. No standardized exercises were performed during this time. The therapy was adapted to the children's individual needs.

### Previous methods used the iES to assess the change in GMFM‐66 scores

Recently, a method of calculating the iES to measure changes in GMFM‐66 over a 6‐month period has been presented. The aim is to assess expected motor changes over 6 months in children with CP in Germany under standard care.^5^ The following formula was used by Duran et al. for this purpose:^5^

$$\begin{array}{c} \text{individual effect size}\\ =\frac{Z_{2}-Z_{1}}{\text{standard deviation of GMFM}-66Z-\text{score difference}\;} \end{array}$$

In the present study, *Z*_1_ is the GMFM‐66 z‐score at baseline and *Z*_2_ the GMFM‐66 z‐score after a 6‐month intensive training programme.^5^ This method can only be used to assess children up to the age of 12 years because the underlying reference centile only goes up to that age.

The iES was evaluated according to typical recommendations for Cohen's *d* (iES ≥0.8: large effect size; 0.5 ≤ iES <0.8: medium effect size; 0.2 ≤ iES <0.5: small effect size; iES <0.2: no effect).^5^

### Establishment of a new tool for evaluating a change in GMFM‐66 scores

Data from children and adolescents aged 3 to 18 years of the follow‐up intervals (M6 to M12 and M4 to M12) were used to establish the new tool. During the follow‐up period, the children received standard care only, and did not participate in another rehabilitation programme.

The difference in the GMFM‐66 score between the M12 and M6 or M12 and M4 measurement time points was calculated and assigned to the age at M6 or M4 respectively. As the follow‐up in the second to fourth rehabilitation years was 2 months longer than in the first year (8 months vs 6 months), their GMFM‐66 score differences were corrected with the factor 6/8. With this correction, all GMFM‐66 score differences were pooled and further analyzed.

A two‐dimensional reference centile was created using the generalized additive model for location, scale and shape (GAMLSS) method (on the basis of the lambda–mu–sigma method).^19^ The explanatory variables were on the one hand age and on the other the GMFM‐66 score at baseline. The gamlss function of the gamlss R package (version 5.4–20) was used to calculate the reference centiles.^19^, ^20^ The degrees of freedom of the explanatory variables ‘GMFM‐66 score at onset’ and ‘age’ were set at 0.5. Normal distribution was used. Both explanatory variables were used to model the parameters of the normal distribution (mean and standard deviation). Instructions for generating centile estimates using two explanatory variables are given in detail by Stasinopoulos et al.^21^ The standard normal distribution of the resulting z‐scores was checked using the Q‐test and the worm plot. The lowest degree of freedom was chosen that still indicated an approximately standard normal distribution of the resulting z‐scores in the Q‐test and the worm plot. With the new reference centiles, it is possible to calculate age‐ and initial‐GMFM‐66 score‐specific z‐scores for a GMFM‐66 score difference, which were calculated for a period of 6 months (after the end of the 6‐month intensive therapy period).

**FIGURE 1:**
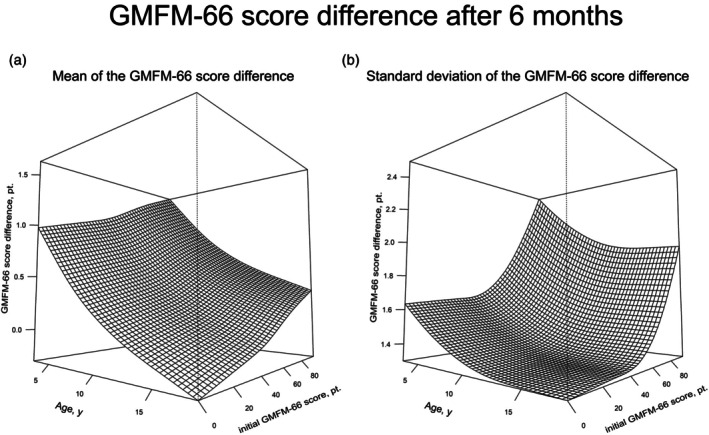
Carpet Diagram: GMFM‐66 score difference after 6 months: three‐dimensional diagrams of (a) the mean GMFM‐66 score difference (after 6 months) as a function of the patient's age (in years) and the initial value of the GMFM‐66 score, and (b) the standard deviation of the GMFM‐66 score difference as a function of the patient's age (in years) and the initial value of the GMFM‐66 score. Abbreviations: GMFM‐66, Gross Motor Function Measure‐66; pt., points.

**FIGURE 2:**
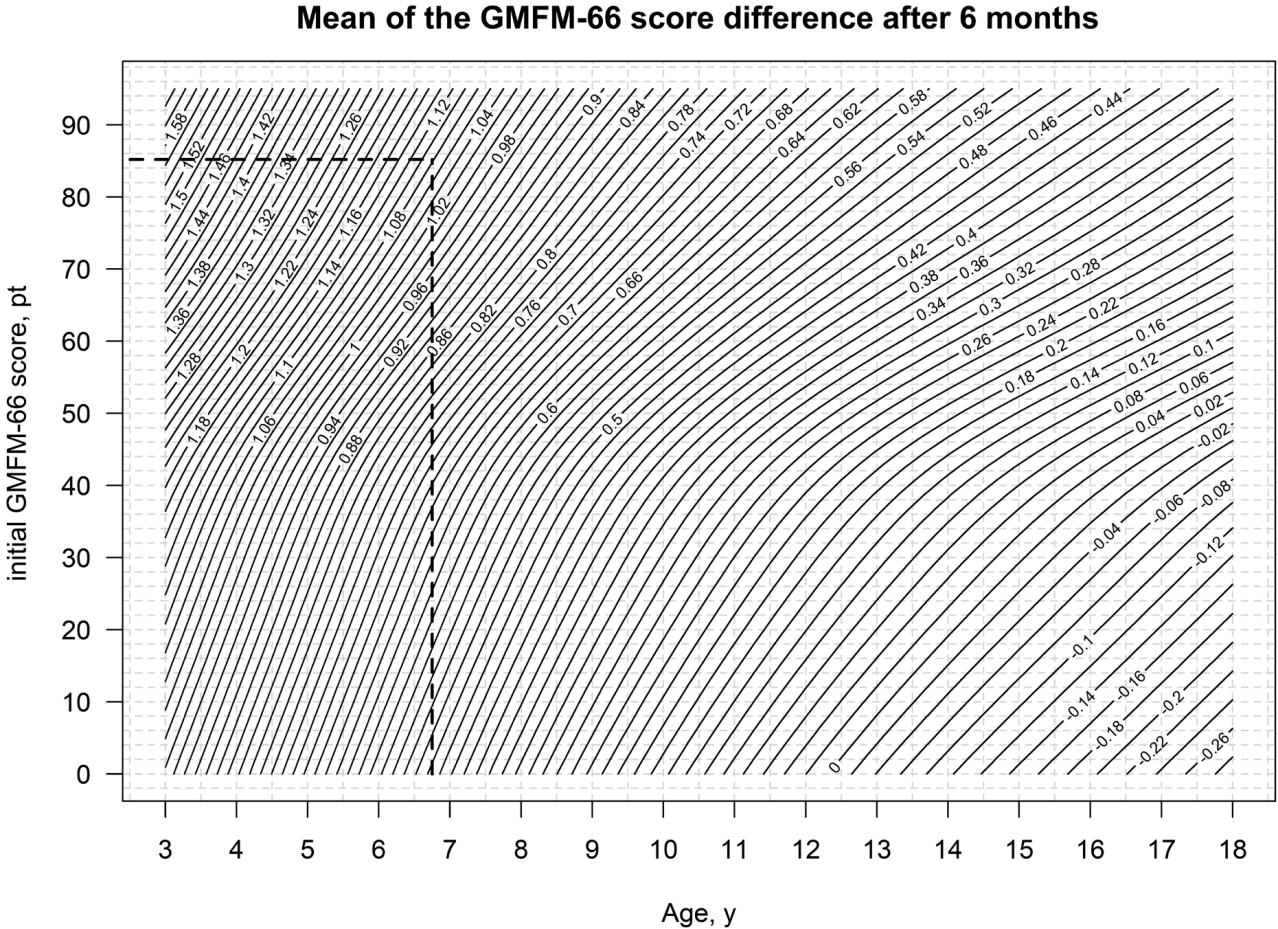
Mean of the GMFM‐66 score difference after 6 months. The figure can be used to identify an individual GMFM‐66 score difference. Abbreviations: GMFM‐66, Gross Motor Function Measure‐66; pt., points.

**FIGURE 3:**
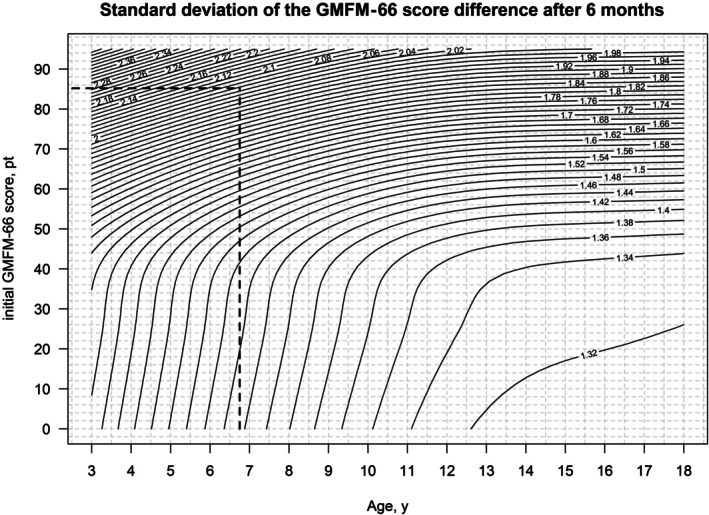
Standard deviation of the GMFM‐66 score difference after 6 months. The figure can be used to identify an individual standard deviation of the GMFM‐66 score difference. Abbreviations: GMFM‐66, Gross Motor Function Measure‐66; pt., points.

The visualization of reference centiles with two explanatory variables is not trivial, as the resulting reference centiles no longer represent a set of curves, but a collection of surfaces (Figure 1). For this reason, representation as contour diagrams (with the ‘contour’ function from R) was used to simplify the representation, in particular for the later applicability of the normative data in everyday clinical practice (Figures 2 and 3). The use of Figures 2 and 3 is described in more detail in the ‘Case report’ section below.

### Validation of the new tool for assessing change in the GMFM‐66 score

Linear regression was used to compare the previous method of Duran et al.^5^ and the novel longitudinal reference centiles. The aim was to determine whether the iES (previous method) could be predicted from the z‐score for the GMFM‐66 score difference (new tool).^5^

The linear regression was calculated using the data from the follow‐up interval (M6–M12 first year; M4–M12 second to fourth year). For better comparability, only data from children under the age of 12 were compared, as the old reference values were only available up to the age of 12 (*n* = 935).

To validate the new tool, the training interval data from the first year of rehabilitation (M0–M6) were used because they included two GMFM‐66 measurements 6 months apart and because these data were neither included in the creation of the new two‐dimensional reference centiles nor in the creation of the linear regression to predict the iES (the two steps of the new tool). Only the data from children under 12 years of age were used (*n* = 876).

Scatter plots and correlation coefficients were used to assess the agreement of the new tool with the existing method by Duran et al.^5^ To compare the agreement in detecting clinically relevant GMFM‐66 differences of the two methods, the sensitivity, specificity, accuracy, area under the receiver operating characteristic curve?, Cohen's kappa, positive predictive value, and negative predictive value were calculated. The new tool represents the ‘test result’, and the true value (clinically relevant change present: yes or no) was determined using the previous method. A change was defined as clinically relevant if the iES (according to the previous method^5^) was less than or equal to −0.5 (deterioration) or greater than or equal to 0.5 (improvement).

### Statistical analysis

RStudio version 2022.02.0 build 443 in conjunction with R version 4.1.3 (R Foundation for Statistical Computing, Vienna, Austria) was used for the analysis. Unless otherwise stated, results are presented as the mean with one standard deviation or frequencies. The significance level was set at *p* < 0.05.

## RESULTS

### Study population

The consort diagrams in Figures S2 and S3 show the composition of the study population. There were 725 GMFM‐66 score pairs from the follow‐up interval of the first rehabilitation year (M6–M12 pairs at a distance of 6 ± 1 months) and 465 GMFM‐66 score pairs from the follow‐up interval of the second to fourth years (M4–M12 pairs at a distance of at 8 ± 1 months) for the establishment of the new two‐dimensional reference centiles. The characteristics of the population used for modelling are detailed in Table 1.

**TABLE 1:** Characteristics of the study population (follow‐up interval).

	M6–M12 pairs^a^	M4–M12 pairs^a^
*n*	725	465
Age, mean (SD), years	7 years 9 months (3 years 10 months)	9 years 11 months (4 years 1 month)
Height, mean (SD), cm	118.6 (20.13)	129.9 (19.4)
BMI, mean (SD), kg/m^2^	15.7 (3.2)	17.0 (4.1)
Female *n*, (%)	303 (41.8)	201 (43.2)
CP subtype, %		
Bilateral	76.7g	78.5
Unilateral	6.9	6.2
Dyskinetic	4.7	3.0
Ataxic	2.6	2.2
Mixed	9.1	10.1
GMFCS level, %		
I	8.1	5.2
II	20.6	23.8
III	38.1	40.8
IV	26.9	27.3
V	6.3	2.8
Time between assessments (SD), years	0.49 (0.06)	0.65 (0.05)

Abbreviations: BMI, body mass index; CP, cerebral palsy; GMFCS, Gross Motor Function Classification System; SD, standard deviation.

^a^ All characteristics were determined at the earlier time point.

See Method for explanation of M0, M4, M6, and M12.

The data of the children for whom longitudinal data were available for the follow‐up interval (M6–M12 and M4–M12 pairs) are listed. The most common CP subgroup was bilateral spastic CP (76.7% and 78.5%), followed by mixed type (9.1% and 10.1%). Children were most commonly classified in GMFCS level III (38.1% and 40.8%).

### Establishment and validation of the new method

The carpet diagram in Figure 1 shows the dependence of (a) the mean and (b) the standard deviation of the GMFM‐66 score differences on the age of the child and the initial value of the GMFM‐66 score in a three‐dimensional illustration.

Figure 2 can be used to estimate the mean and Figure 3 can be used to estimate the standard deviation of the GMFM‐66 score difference depending on the individual's age and initial GMFM‐66 score. The dashed black lines show the application of the contour diagram using a case study which is explained below. Both contour diagrams without the drawn case study can be found in Figures S4 and S5.

**FIGURE 4:**
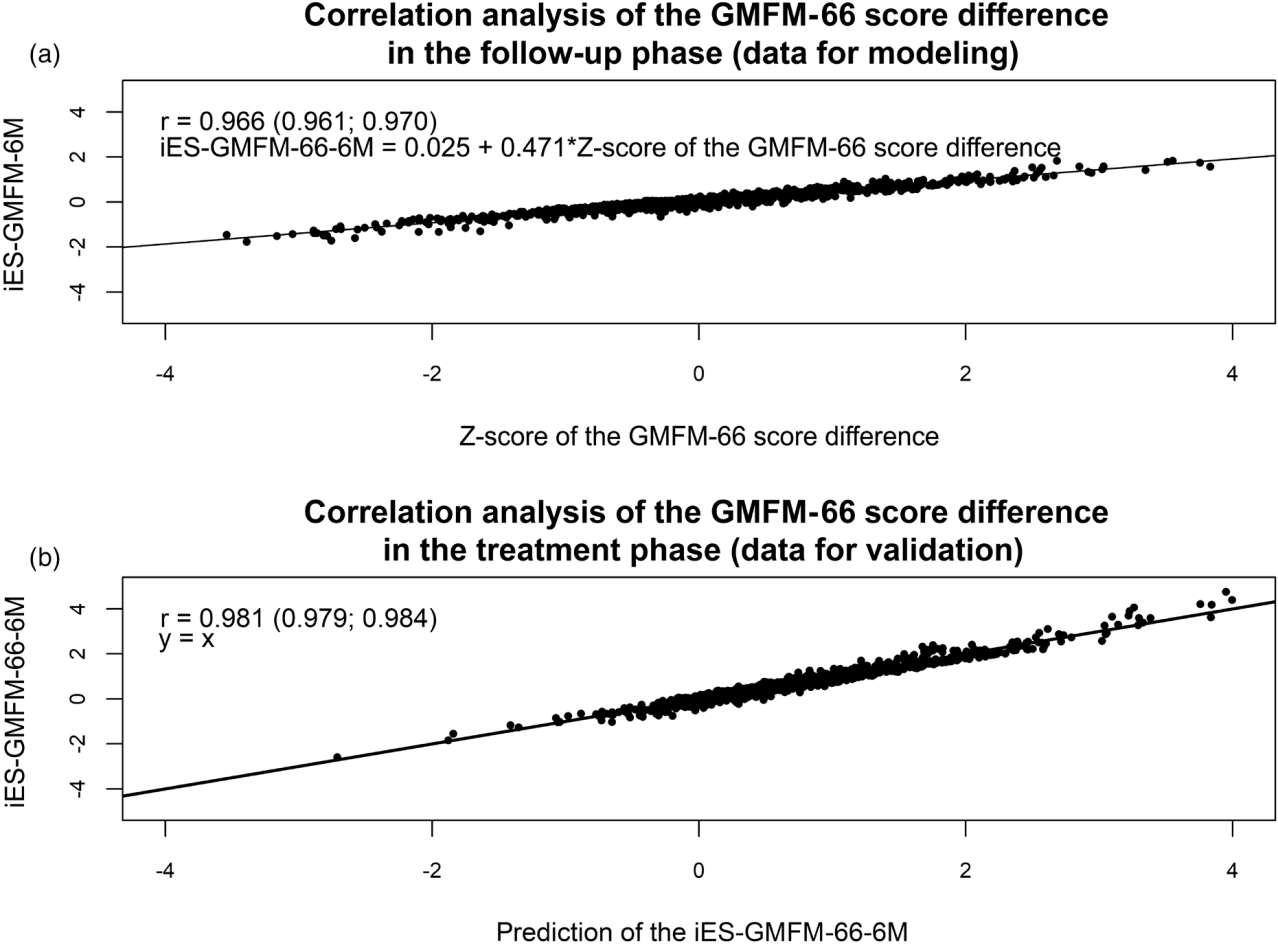
Correlation analysis of the GMFM‐66 score difference in (a) the follow‐up phase (data for modelling) and (b) the treatment phase (data for validation). Abbreviations: GMFM‐66, Gross Motor Function Measure‐66; iES, individual effect size.

Figure 4a shows scatter plots of the iES calculated with the previous method and the z‐scores of the GMFM‐66 score differences based on the new tool (Pearson's correlation coefficient *r* = 0.966, *p* < 0.001). The linear regression between the two variables is also shown.

To validate the new tool, the iES of the GMFM‐66 difference after the training interval (M0–M6) was determined (Figure 4b), using the previous method (*y*‐axis) and using the new tool (*x*‐axis). Pearson's correlation coefficient of both values was *r* = 0.981 (*p* < 0.001).

### Prediction of a clinically relevant change in motor function with the new tool

Table 2 shows various statistical quality criteria (area under the receiver operating characteristic curve, positive predictive value, negative predictive value, specificity, sensitivity, accuracy, and Cohen's kappa) of the new tool for detecting a clinically relevant change in the GMFM‐66.

**TABLE 2:** Agreement between the described two methods for predicting clinically relevant change in gross motor function.

Parameter	Goodness of the new method to predict clinically relevant change in gross motor function defined by iES	
	At least moderate improvement	At least moderate deterioration
	Predicted iES ≥ 0.5	Predicted iES ≤ −0.5
Accuracy	0.936 (0.918; 0.951)	0.981 (0.969; 0.989)
AUC	0.991 (0.987; 0.995)	0.990 (0.982; 0.999)
Cohen's kappa	0.872 (0.805; 0.938)	0.702 (0.639; 0.766)
PPV	0.905 (0.873; 0.931)	0.955 (0.771; 0.998)
NPV	0.967 (0.945; 0.981)	0.981 (0.970; 0.989)
Sensitivity	0.963 (0.940; 0.979)	0.568 (0.395; 0.729)
Specificity	0.913 (0.884; 0.937)	0.999 (0.994; 1.0)

True moderate improvement (deterioration) was defined as iES ≥ 0.5 (≤ −0.5).

Abbreviations: AUC, area under the receiver operating characteristic curve; iES, individual effect size; NPV, negative predictive value; PPV, positive predictive value.

## DISCUSSION

With the new reference centiles, it is possible to calculate age‐ and initial‐GMFM‐66 score‐specific z‐scores for a GMFM‐66 score difference, which were calculated for a 6‐month period. The novel reference centiles demonstrate a high degree of concordance with the previously described method of calculating the iES by Duran et al.^5^ Both methods agree in detecting a clinically relevant improvement in 93.6% and a deterioration in 98.1% of cases (Table 2). The z‐score of the GMFM‐66 score difference using the new tool and the iES by the previous method show a very high correlation (*r* = 0.981) (Figure 1b). The highly consistent result is remarkable because the previous method by Duran et al.^5^ was based on cross‐sectional data, whereas the new tool is based on longitudinal data.

Rosenbaum et al. presented longitudinal GMFM‐66 reference values for each of the five GMFCS levels for children with CP between the ages of 1 year and 13 years.^13^ Hanna et al.^12^ and Smits et al.^11^ also presented longitudinal GMFM‐66 centile curves, but these were subdivided by severity according to the GMFCS.^11^, ^12^ Palisano et al. demonstrated a correlation between the results of the GMFM‐66 and the GMFCS level. It could be shown that a patient classified in GMFCS level I could achieve a higher score on the GMFM‐66 than a patient in GMFCS level V, because they were able to perform more test items correctly.^22^ However, Duran et al.^5^ showed that it is sufficient to construct a single GMFM‐66 centile curve for all GMFCS levels, taking into account age and initial GMFM‐66 score. In simple terms, it can be explained as follows. The reference centiles delineated by Duran et al. comprise a multitude of centiles. The centiles in the low range (e.g. the third centile or z‐score = −1.88) demonstrate a flat curve, with the result that only a very low GMFM‐66 score is attained at the age of 12 years. The 97th centile (equivalent to a z‐score of +1.88) demonstrates a remarkably steep course, attaining a notably elevated GMFM‐66 score by the age of 12 years. Consequently, the arbitrary classification of CP severity into five degrees is rendered obsolete in the present context.^5^ To prove this, Duran et al. created a reference curve with all GMFM‐66 scores, and the children were then classified using the GMFCS level (method A). In method B, a reference curve was created for each GMFCS level (five in total). Both methods were compared with each other in terms of their test quality. It was shown that one GMFM‐66 reference curve (method A) could predict a GMFM‐66 change just as well as five different reference curves based on GMFCS levels (method B).^5^ The centile curves of Duran et al. agreed very well with the reference curves of Hanna et al. and Smits et al.^5^ It therefore seems clearer in everyday clinical practice to use only one GMFM‐66 centile curve. Another advantage is that with the iES of the GMFM‐66, changes can be determined after a period of 6 months.^5^

The advantage of the new tool of the present study is that it can be used on children from 3 to 18 years of age and is not limited to an age of 11 years as in the reference curves of Duran et al.^5^ and Rosenbaum et al. (1–13 years).^13^ The GMFM‐66 reference curve in this study has two explanatory variables (age and initial GMFM‐66 score). Their use differs from the usual reference centiles used in everyday clinical practice. Therefore, their use is demonstrated in a clinical example, as follows.

### Case report

A child with CP had a GMFM‐66 score of 85.2 at the age of 6 years 8 months. They participated in the rehabilitation programme and had a GMFM‐66 score of 89.7 after 6 months. The question is, had the child benefited from the rehabilitation programme?

The first step in the new tool is to calculate the z‐score for the GMFM‐66 score difference. To do this, the mean and standard deviation must be estimated using Figures 2 and 3. For this, the age at the start of the observation period is plotted on the *x*‐axis and the GMFM‐66 score on the *y*‐axis. The mean (1.08; Figure 2) and standard deviation (2.04; Figure 3) can then be read from the contour diagram. The observed change in GMFM‐66 score is 89.7–85.2 = 4.5.

The z‐score is calculated using the formula z‐score = $\frac{\text{GMFM}-66\;\text{score}\_\text{difference}-\text{mean}}{\text{standard deviation}.}$ and is thus (4.5–1.08)/2.04 = 1.68.

In the second step, the iES is calculated using the linear regression formula from Figure 3a (iES = 0.025 + 0.471 × z‐score)

Here, iES = 0.025 + 0.471 × 1.68 = 0.82. Since the iES is assessed in the same way as Cohen's *d*, the result is that the observed difference in GMFM‐66 score after participation in the rehabilitation programme indicates a large, clinically relevant effect (considering the expected motor development under standard care in 6 months). In the literature, clinical relevance is considered from an effect size of 0.5.^5^ This clinical example was also used in the presentation of the previous method. There, an iES of 1.04 was calculated, meaning that a large effect size was also found with the previous method by Duran et al.^5^

The mean and the standard deviation can also be read from the tabular reference centiles (Tables S1 and S2): the GMFM‐66 is rounded to a whole number (here: 85) and the age is the closest to the entry (here: 6.5 years). This gives a mean of 1.112 and a standard deviation of 2.043. As described above, this gives a z‐score for the GMFM‐66 score difference of 1.66 and for the iES of 0.81. The advantage of the tabular reference centiles is that they can be processed with little effort using a script, for example for a patient's electronic record.

In addition, it was possible to examine the iES of all participants in the rehabilitation programme aged 3 to 18 years and to determine a mean iES, which was 0.65 (95% confidence interval 0.60–0.71, *n* = 1019), and thus to assess the effectiveness of the rehabilitation programme itself. The iES of children under 12 years (mean iES = 0.66 [0.60–0.72], *n* = 895) and over 12 years (mean iES = 0.62 [0.46–0.77], *n* = 124) did not differ significantly (*p* = 0.538).

### Limitations

This study had several limitations. The data used in the creation of the referenced centiles originated from children and adolescents who participated in a rehabilitation programme (selection bias). In the follow‐up phase, however, no specific therapeutic interventions were administered by the rehabilitation programme. The children received the standard care that is provided to all children in Germany with CP, which included approximately twice‐weekly physiotherapy sessions (for 30 minutes). As the reference values are designed to evaluate the change in the GMFM‐66 over a 6‐month period, rather than the measurement at a single point in time, they can be transferred to other children with CP in Germany.

Most children and adolescents in the study were classified in GMCFS levels II to IV. Children classified in GMFCS levels I and V were underrepresented because they had too mild or severe motor symptoms to participate in the rehabilitation programme.

The study was a retrospective data analysis of prospectively collected data. There were 1817 missing values in the first year of rehabilitation and 905 missing values in the second to fourth years of the rehabilitation programme, because in some cases fewer than 60 test items were performed.

All CP subgroups and GMFCS subgroups were represented in the study. Most of the children had the spastic subtype, which is the most common form of CP in Europe.^1^

It should be noted that the two GMFM‐66 measurements should be taken approximately 6 months apart so that the change in the observation period can be adequately assessed. In this study, we used a cut‐off of ±1 month. If the period is significantly longer than 6 months, the calculated iES would overestimate the motor development; if it is significantly shorter, the motor development would be underestimated.

The reference values presented here only refer to the evaluation of a 6‐month observation period. Future studies are needed to consider a longer observation period.

## CONCLUSION

In conclusion, the presented reference centiles are a new method to assess changes in the GMFM‐66 score over a period of 6 (±1) months, for example to evaluate the therapeutic impact of an intervention. It shows remarkable agreement with the previously described method (calculation of the iES).^5^ The advantage of the new method is that changes in motor function of children and adolescents from three to 18 years of age can now be detected and that the refence centiles are not limited to an age of 12 years.

## FUNDING INFORMATION

This study received no external funding.

## CONFLICT OF INTEREST STATEMENT

The authors have stated that they had no interests that might be perceived as posing a conflict or bias.

## Supporting information


**Figure S1:** Rehabilitation program.

**Figure S2:** Consort diagram for first rehabilitation year.

**Figure S3:** Consort diagram for second to fourth rehabilitation years.

**Figure S4:** Mean of the GMFM‐66 score difference after 6 months without case report.

**Figure S5:** SD of the GMFM‐66 score difference after 6 months without case report.

**Table S1:** Mean of the GMFM‐66 score difference after 6 months.

**Table S2:** SD of the GMFM‐66 score difference after 6 months.

## Data Availability

Data available on request due to privacy/ethical restrictions.
