# Supplementary figures and images for: Longitudinal reference centiles for the Gross Motor Function Measure‐66 in children and adolescents with cerebral palsy
Source: Dev Med Child Neurol. 2025 Aug 5;68(2):218–26. doi: 10.1111/dmcn.16455 (PMC12766559; doi:10.1111/dmcn.16455)

# Rehabilitation program „Auf die Beine“

## First year

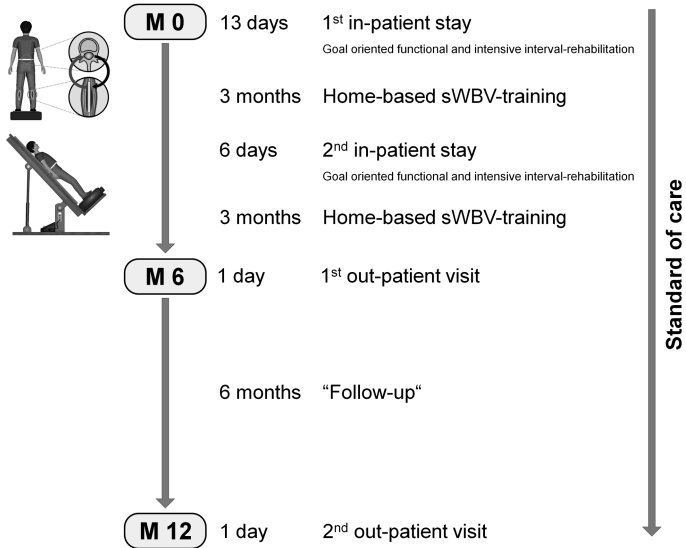

## Second-fourth year

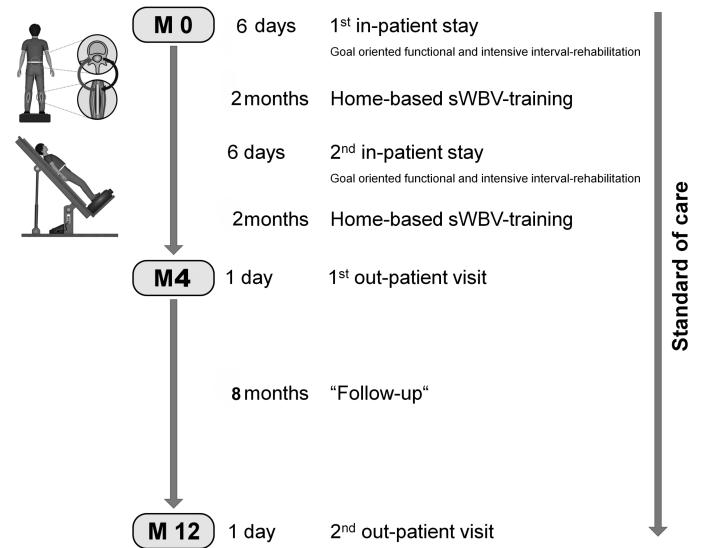

Supplement: Supplementary file 1 — Figure S1: Rehabilitation program. [file DMCN-68-218-s002.pdf]

First year of the rehabilitation program

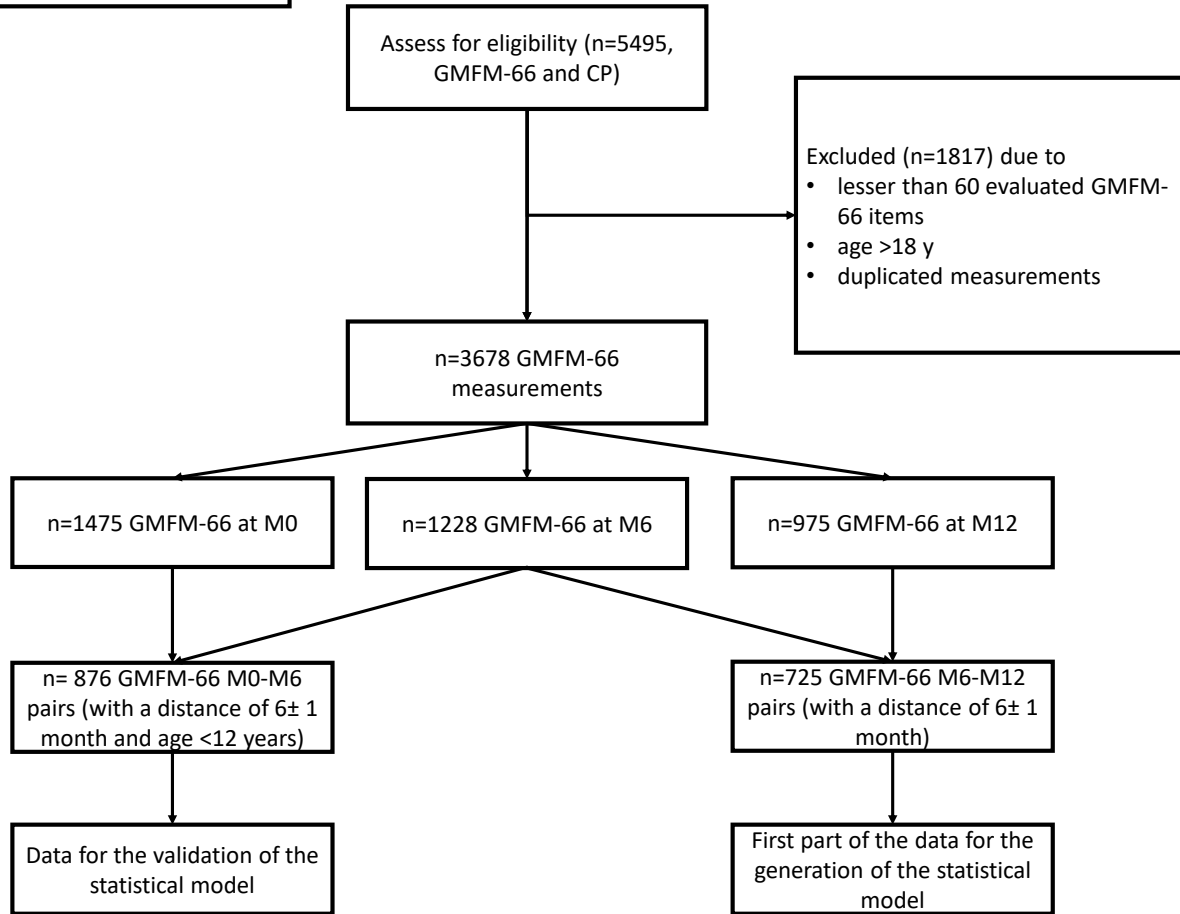

Supplement: Supplementary file 2 — Figure S2: Consort diagram for first rehabilitation year. [file DMCN-68-218-s006.pdf]

Second to forth years of the  
rehabilitation program

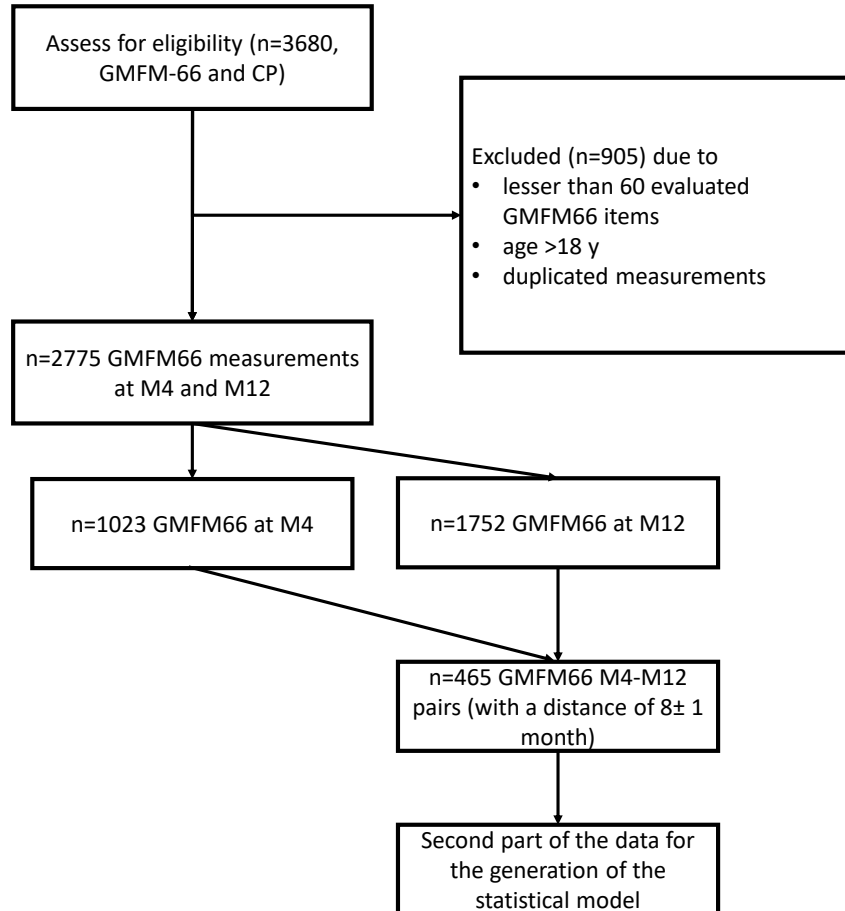

Supplement: Supplementary file 3 — Figure S3: Consort diagram for second to fourth rehabilitation years. [file DMCN-68-218-s004.pdf]
